# Comparison of anti-HBV regimen with or without adefovir on hepatocellular carcinoma development of Chronic hepatitis B patients with compensated cirrhosis: a retrospective cohort study

**DOI:** 10.1186/s13027-018-0189-2

**Published:** 2018-05-22

**Authors:** Jing Sun, Yanfang Li, Yanna Wang, Yanyan Liu, Youde Liu, Xiumei Wang

**Affiliations:** 1 The seventh department, Yantai Infectious Disease Hospital, Yantai, Shandong 264001 People’s Republic of China; 2 Central laboratory, Anhui Medical University, Hefei, Anhui 230601 People’s Republic of China; 3 Department of oncology, Yantai Yuhuangding Hospital, Yantai, Shandong 264000 People’s Republic of China

**Keywords:** Adefovir, Chronic hepatits B, Hepatocellular carcinoma, Cirrhosis, Nucleus(t)ide analogues

## Abstract

**Background:**

The impact of different anti-virus regimens on prognosis of Chronic hepatitis B (CHB) related cirrhosis remains to be explored. We aim to investigate whether CHB-related HCC patients receiving nucleoside analogue regimen or not have a different prognosis.

**Methods:**

242 CHB-related compensated cirrhosis patients from 2008 June to 2011 December were included in our study and attributed into groups based on their anti-virus regimens containing adefovir (ADV) or not. The clinical parameters and virological response between ADV-containing regimen group and non-ADV containing regimen groups were reviewed and compared. The risk of hepatocellular carcinoma (HCC) development were analyzed and compared between two groups.

**Results:**

127 patients received anti-virus regimen containing ADV and 115 patients received anti-virus regimen without ADV. The cumulative risk of HCC development among patients treated with ADV-contained therapy was significantly lower than that observed in patients with non-ADV-contained therapy (*p*<0.05). Multivariate analysis indicated that ADV-containing regimen treatment was significantly associated with lower probability of HCC development, (hazard ratio, 0.18; 95% confidence interval range, 0.07-0.45, *p*<0.05).

**Conclusion:**

Both anti-virus regimens were effective in reducing serum HBV DNA. Regimen containing ADV decreased the incidence of HCC development in CHB patients with compensated cirrhosis.

## Background

Hepatocellular carcinoma (HCC) is among the most common malignancies of high morbidity and mortality, especially in China [1, 2]. Chinese HCC patients accounted for majority of HCC-related mortality in the world [1–4]. Factors associated with the development of HCC include: hepatitis C virus infection, alcholic hepatic diseases, smoking and *et al*, among which chronic hepatits B (CHB) infection is highly related to HCC development [5–8]. CHB-related liver cirrhosis and HCC patients is predominant in Chinese HCC patients, for hepatits B virus (HBV) infection is highly prevalent in China [9, 10].

Serum HBV-DNA level is a key predictor for liver cirrhosis and it has been proved to be correlated with cirrhosis progression [11]. The contribution of persistent HBV replication to liver cirrhosis and HCC in CHB patients has been determined in several studies [12–14]. Thus, sustained suppression of HBV replication is regarded as a critical therapeutic strategy to reduce liver cirrhosis or HCC development [15]. Nucleos(t)ide analogues (NAs) have been determined to be highly effective for suppressing HBV replication [16], as well as regression of cirrhosis and reduction of HCC incidence among CHB patients [15, 17–19].

Surgical resection is still regarded as main curative therapy for localized HCC for it may provide probability of disease-free survival in HCC patients [20]. Sustained HBV replication has a strong association with recurrence in CHB-related HCC patients after surgery, so anti-HBV treatment is necessary for CHB-related HCC patients [21]. It has been reported that NAs can provide additional benefits for CHB-related HCC patients receiving local treatment [22–24].

Although both monotherapy and combination regimen of NAs have been proved to be effective in CHB-related HCC patients [22–24], it remains to be explored that whether there is any discrepancy among each regimen on their potential benefits to CHB-related compensated cirrhosis. Recently, it is reported that nucleutide analogues, rather than nucleuside analogues could induce the expression of IFN-λ [25], which reminded us whether there would be any difference regarding the prognosis of CHB-related compensated cirrhosis patients with different NAs regimens. Thus, we designed a retrospective cohort study to explore the difference of potential impact on CHB-related cirrhosis patients with different NAs regimens.

## Methods

### Patients and study design

All CHB patients were diagnosed with compensated liver cirrhosis and received anti-HBV treatment in Yantai Infectious Disease Hospital and Yantai Yuhuangding hospital (Shandong, China). Inclusion criteria were: (1) Diagnosed as HBV infection with compensated liver cirrhosis; (2) Child-Pugh scoring ≤ 9; (3) Valid clinical characteristics and laboratory outcomes. The exclusion criteria were: (1) Hepatocellular carcinoma; (2) HCV and HDV co-infection; (3) Alcoholic hepatic diseases; (4) Schistosomiasis; (5) Invalid clinical characteristics and laboratory outcomes; (6) Anti-virus regimen switching from non-ADV containing to ADV containing *vice versa* or received interferon treatment. 339 patients were included into this retrospective study and 97 patients were excluded due to HCV co-infection (*n*=12), HDV co-infection (*n*=3), alcoholic hepatic diseases (n=6), schistosomiasis (*n*=4), invalid data (*n*=6), regimen switching due to virologic breakthrough (*n*=36), interferon treatment (*n*=10) and lost (*n*=20). The final patients included in this study were 242.

This study was conducted under compliance with the Declaration of Helsinki and were approved by *the Human Ethics Committee of Yantai Infectious Disease Hospital and the Human Ethics Committee of Yantai Yuhuangding hospital.*

### Diagnosis

All patients were histologically confirmed with cirrhosis using specimen from liver biopsy or contrast-enhanced CT. HBV infection were diagnosed by positive serum viral marker and elevated serum HBV-DNA level (>1000 copies/mL during two consecutive detection). Quantification of serum HBV DNA was measured by real-time quantitative PCR assay with Roche LightCycler (Roche Diagnostics, Basel, Switzerland) and suitable reagents (PG Biotech, Shenzhen, China), of which the lower limit of quantification is 1000 copies/mL and the linear range was between 1120 and 6.69 log copies/mL. Contrast-enhanced CT, ultrasonography or liver biopsy were conducted to screen HCC recurrence during follow-up. Child-Pugh scoring was applied for consideration of prognosis as previously reported [26].

### HBV treatment

ADV-containing regimen included ADV monotherapy (*n*=13) or combined with lamivudine (LAM) (*n*=95) and entecavir (ETV) (*n*=19). Non-ADV containing regimen included LAM (*n*=42), ETV (*n*=61) or telbivudine (LdT) (*n*=12) monotherapy. The dosage of NAs in all patients were 10 mg per day for ADV, 100 mg per day for LAM, 0.5 mg per day for ETV and 600 mg per day for LdT. In non-ADV containing group, 11 patients receiving LAM and 12 patients receiving LdT were switch to ETV due to virologic breakthrough.

### Statistics

Continuous variables were expressed as mean ± SD with normal distribution and median (range) without normal distribution. The comparison of continuous variables with or without normal distribution was analyzed with Student *t* test and Wilcoxon rank test, respectively. Chi-square and Fisher’s test were applicated for analysis of categorical variables. *P*<0.05 was regarded as statistically significant. The univariate analysis was conducted through Kaplan-Meier statistics and Log-rank test. Multivariate analysis was assessed with Cox regression test. Variables with *p*<0.05 were employed into the Cox regression model. *P*<0.05 was considered as statistically significant. Statistics analysis was conducted with SPSS (version 16.0, SPSS Inc., Chicago, IL, USA) software package. Figures were made with GraphPad Prism 5 software.

## Results

### The baseline characteristics

242 CHB patients with compensated cirrhosis were distributed to ADV-containing group (*n*=127) and non-ADV-containing group (*n*=115) according to their anti-virus regimen. The average age of ADV-containing group and non-ADV-containing group were 50±12 and 50±10, respectively. Male patients were predominant in both groups: ADV-containing group (*n*=114, 89.8%) and non-ADV-containing group (*n*=98, 85.2%). Median total bilirubin was 16.1 and 14.4 μmol/L in ADV-containing group and non-ADV-containing group, respectively. The percentage of patients with Child-Pugh scoring A was 96.1% (*n*=122) in ADV-containing group and 95.6% (*n*=110) in non-ADV-containing group (Table 1).

**Table 1:** Baseline characteristics of CHB-related compensated cirrhosis

	ADV-containing	Non-ADV-containing	*p*-value
	(n=127)	(n=115)	
Age, (mean ± SD)	50 ± 12	50 ± 10	0.62
Gender			0.28
Male	114 (89.8%)	98 (85.2%)	
Female	13 (12.4%)	17 (9.7%)	
HBV DNA (log10 copy/mL, mean ± SD)	3.93 ± 1.31	3.88 ± 1.18	0.76
HBeAg			0.92
Positive	92 (72.4%)	84 (73.0%)	
Negative	35 (27.6%)	31 (27.0%)	
ALT (U/L, mean ± SD)	98.3 ± 23.6	96.3 ± 24.4	0.34
Total bilirubin (μmol/L), median (range)	16.1 (5.7-65.0)	14.4 (5.5-39.8)	0.11
ALB (g/L), median (range)	4.5 (3.8-6.3)	4.0 (2.9-6.1)	0.09
AFP, ng/mL, median (range)	4.91 (1.21-28)	5.41 (1.16-31)	0.08
PLT, 10^9/L, median (range)	186.31 (97-231)	178.44 (103-241)	0.13
Child-Pugh score			0.87
A	122 (96.1%)	110 (95.6%)	
B	5 (3.9%)	5 (4.4%)	

*HBeAg* hepatitis B e antigen, *AFP* α-fetoprotein, *ALB* albumin, *PLT* platelet, *SD* standard deviation

No significant difference of virological characteristics between two groups (Table 1). Serum HBV-DNA level in ADV-containing group and non-ADV-containing group were 3.93 ± 1.31 (log10 copy/mL, mean ± SD) and 3.88 ± 1.18 (log10 copy/mL, mean ± SD), respectively. The percentage of positive HBe antigen were 72.4% (*n*=92) in ADV-containing group and 73.0% (*n*=84) in non-ADV containing group. 37% (*n*=47) patients in ADV-containing group and 26.9% (*n*=31) in non-ADV containing group were HBe antibody positive. 11 patients in ADV-containing group and 9 patients in non-ADV containing group were HBsAg negative.

### Virological, serological and biochemical response

All patients achieved virological response by 48 weeks after NAs regimens treatment (HBV DNA < 1000 copies/mL). The median NAs duration were 57 months in ADV-containing group and 51 months in non-ADV containing group. 13 patients receiving ADV had LAM add-on for sustained positive serum HBV DNA. 11 patients receiving LAM and 12 patients receiving LdT in non-ADV-containing group were switched to receiving ETV by 24 weeks. 32 patients in ADV-containing group and 16 patients in non-ADV-containing group experienced virological breakthrough due to poor adherence (HBV DNA >1000 copies/mL). 64.1% patients (*n*=59) in ADV-containing group and 51.2% patients (*n*=43) in non-ADV containing group achieved HBeAg seroconversion. All patients achieved ALT normalization by 96 weeks.

### The development of HCC

**Fig. 1:**
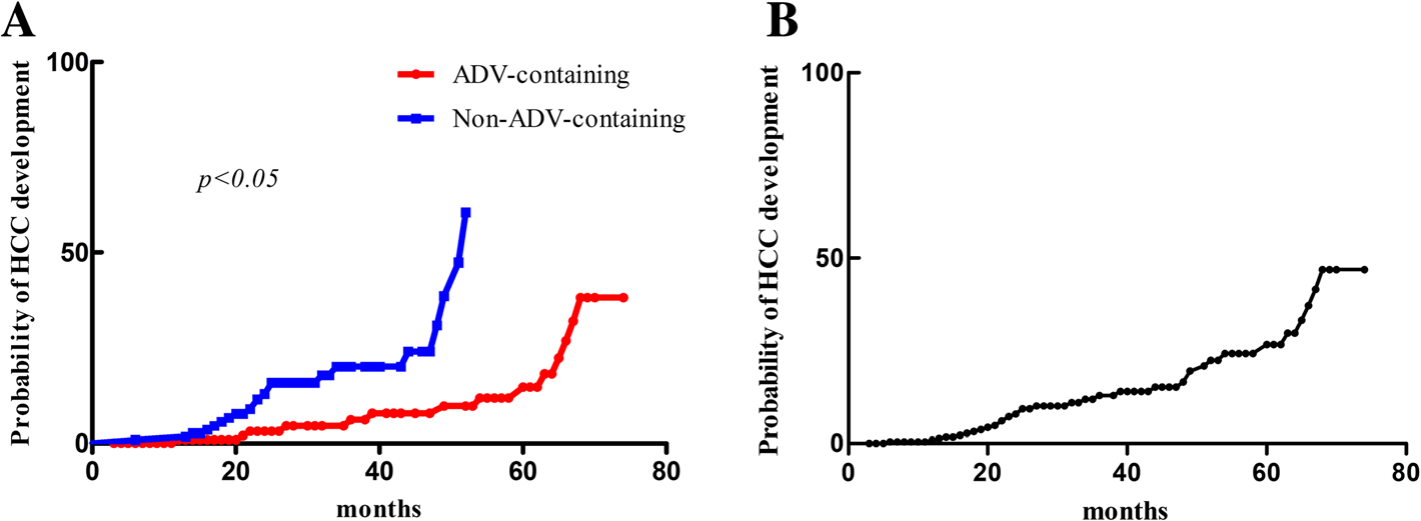
The cumulative probability of HCC development. **a**: the comparison of cumulative HCC development probability between ADV-containing group (red) and non-ADV-containing group (blue). X-axis represented time (month), Y-axis represented probability of HCC development; **b**: the cumulative probability of HCC development in all patients

Overall, 14.5% patients (*n*=35) developed HCC during follow up. 11.0% patients (*n*=14) in ADV-containing and 18.3% patients (*n*=21) in non-ADV-containing group developed HCC. 5-year cumulative probability of HCC development in all patients were 26.7%. The cumulative probability of HCC development in ADV-containing group (*n*=127) was significantly lower than it in non-ADV-containing group (*n*=115) (hazard ratio, 0.18; 95% confidence interval range, 0.07-0.45, *p*<0.05) (Fig. 1a). In order to identify the potential factors related to the probability of HCC development, we also conduct univariant and multi-variant analysis to investigate the association between baseline characteristics and HCC development. Univariant and multi-variant analysis indicated that ADV-containing regimen treatment was independently associated with lower HCC development rate (hazard ratio, 0.18; 95% confidence interval range, 0.07-0.45, *p*<0.05) (Table 2).

**Table 2:** Univariate and multivariate analysis of baseline characteristics with HCC development

	univariant analysis	*p*-value	multivariant analysis	*p*-value
	HR (95% CI)		HR (95% CI)	
Age	1.00 (0.99–1.01)	NS		
Gender:male/female	3.11 (0.99-9.72)	NS		
Child-Pugh score: A/B	0.48 (0.21-1.10)	NS		
ADV-containing/Non-ADV-treatment	0.18 (0.09-0.43)	<0.05	0.18 (0.07-0.45)	<0.05
Total bilirubin: < 24/≥ 24 (μmol/L)	0.52 (0.19-1.41)	NS		
HBV DNA: <4/≥4 (log copies/ mL)	0.85 (0.41-1.76)	NS		
HBeAg: Positive/negative	1.16 (0.54-2.50)	NS		

*HBeAg* hepatitis B e antigen

## Discussion

Our study demonstrated that anti-virus regimen containing ADV provide a lower probability of HCC development than regimen without ADV in CHB related compensated cirrhosis patients. It has been determined that sustained suppression of HBV replication is fundamental for CHB patients and delays hepatic diseases progressing to end stage liver diseases, such as decompensated cirrhosis and HCC [27, 28]. However, regarding the access to anti-virus agents, which regimen is suitable for patients with cirrhosis is not determined. Our study provided evidence for appropriate anti-virus treatment to management of CHB related compensated cirrhosis patients.

Sustained suppression of HBV replication is the fundamental principle for anti-HBV therapy [29, 30]. The median NAs duration in our study was up to four years, to which was contributed by local reimbursement policy. In our study, all patients had ALT normalization and negative serum HBV DNA during long term NAs duration. However, 13 patients with ADV monotherapy were still serum HBV DNA positive after 56 weeks treatment. NAs monotherapy with agents of low resistance barrier is not suitable for long term treatment.

Recent studies revealed that nucleutide analogue rather than nucleoside analogue provide additional effect to induce expression of interferon-λ3 [25], since interferon-λ3 has been demonstrated to be involved in modulation of immunity during virus infection or autoimmune diseases [31]. Inflammation is determined to have a strong association with carcinogenesis and recurrence of HCC [32]. In our study cumulative probability of HCC development in ADV containing group is significantly lower than non-ADV containing group, which might be caused by hypothesis above. But the mechanism requires further clinical evidence.

The main flaw in our study is the limitation to serum HBV marker quantification, which might reveal the potential mechanism of our outcomes. The association between nucleotide analogues treatment and HBsAg reduction have been proved [25], as well as the relationship between HCC and HBsAg [33]. In our study, serum HBsAg titer measurement is limited to patients as high cost and poor accessibility during patient recruitment, which will be included in further study. As a retrospective study, the bias in data collection and poor compliance to administration of patients also limited further analysis since a majority of patients included in our study received anti-virus treatment outside of hospital. Thus, a prospective randomized clinical trial to compare anti-virus regimens may provide a better analysis and solid evidence.

## Conclusion

Both anti-virus regimens were effective in reducing serum HBV DNA. Regimen containing ADV decreased the incidence of HCC development in CHB patients with compensated cirrhosis.

## Abbreviations

ADV: Adefovir
ALT: Alanine aminotransferase
CHB: Chronic hepatits B
ETV: Entecavir
HBeAg: Hepatitis B e antigen
HBsAg: Hepatits B surface antigen
HBV: Hepatitis B virus
HCC: Hepatocellular carcinoma
LAM: Lamivudine
LdT: Telbivudine
NAs: Nucleus(t)ide analogues
SD: Standard deviation
